# Mediastinal bronchogenic cyst presenting with dysphagia and back pain

**DOI:** 10.4103/0970-2113.63612

**Published:** 2010

**Authors:** Manish Kumar Tiwari, Rajkumar Yadav, Rajendra Mohan Mathur, Chandra Prakash Shrivastava

**Affiliations:** *Department of C.T.V.S., S.M.S. Medical College & Hospital, Jaipur, Rajasthan, India*

**Keywords:** Back pain, bronchogenic cyst, dysphagia, mediastinal cyst

## Abstract

The presentation of the bronchogenic cyst is variable, making pre-operative diagnosis difficult. Majority of them are either asymptomatic or discovered incidentally. The most common presenting symptoms are cough, fever and dyspnea. We discuss the case of a large bronchogenic cyst in the posterior mediastinum causing oesophageal compression and impinging on the left atrium. The patient presented with dysphagia and back pain and was extensively investigated by various physicians before being diagnosed as having bronchogenic cyst. We concluded that the backache was due to stretching of nerves in the parietal pleura. This case demonstrates the need for detailed investigations prior to treatment of patients with such symptom complex as a bronchogenic cyst may be the cause of such symptoms.

## INTRODUCTION

Bronchogenic cyst, though rare, is the most common primary cyst of the mediastinum and accounts for 6.3% of primary mediastinal masses and 34% of the cysts. Two-thirds of the patients are asymptomatic. This case presented with dysphagia and back pain which is a rare and clinically confusing presentation for this entity. A thorough review of literature showed very few cases with such rare presentation.

## CASE REPORT

**Figure 1:**
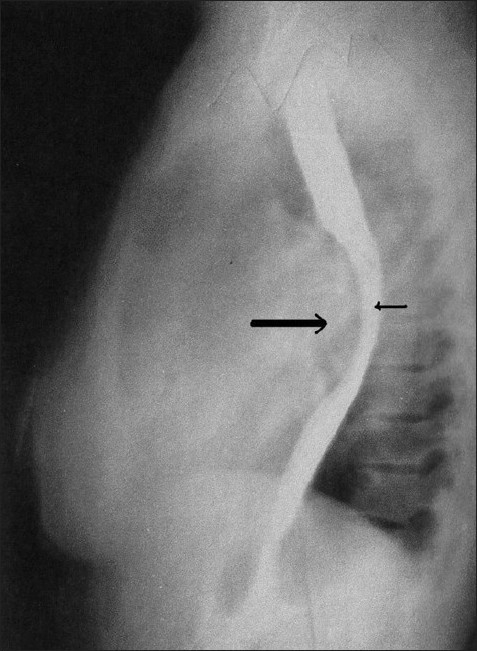
Barium swallow shows large extrinsic compression (large arrow) anteriorly on mid-esophagus causing narrowing of the region (small arrow)

A 35-year-old female patient presented with a history of dull, aching pain in the inter-scapular region and dysphagia for solid diet of one-month duration. Patient was evaluated by a medical gastro-enterologist; Barium swallow, Oesophago-gastro-duodenoscopy (OGD) and USG abdomen were done apart from the routine investigations. Hematology was unremarkable, except, raised ESR of 72 mm/first hr. An ultrasonography of the abdomen showed solitary 2.5 cm large gall bladder calculi. An OGD revealed normal oesophageal mucosa with signs of extrinsic compression over anterior oesophageal wall and mild gastritis. Barium swallow showed large extrinsic compression anteriorly on mid-oesophagus causing narrowing of the region [Figure 1] possibly due to enlargement of the left atrium (LA). ECG showed non-specific ST-T changes.

Patient was referred to a cardiologist for evaluation prior to cholecystectomy. A 2D-echocardiography showed normal heart with a large cystic structure, situated behind LA, with mild tricuspid regurgitation.

**Figure 2:**
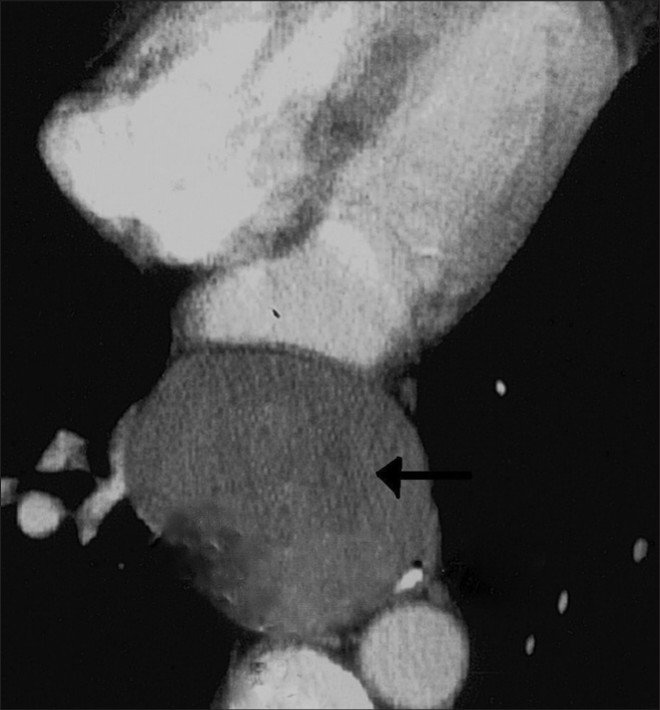
Contract enhanced chest CT scan shows 9.5 × 6.5 × 6 cm well defined homogenous cystic lesion (arrow) having attenuation values of 0-20 HU in the middle mediastinum encroaching upon the posterior mediastinum and compressing the esophagus

The patient was admitted in the cardio-thoracic unit. Chest X-ray showed well defined round mass lesion with superior displacement of bilateral principle bronchi. X-ray of vertebral column showed no abnormality. Post-contrast helical chest CT scan showed a 9.5 × 6.5 × 6 cm well defined homogenous cystic lesion having attenuation values of 0-20 HU beginning at the carina and extending downwards in the middle mediastinum [Figure 2]. It caused displacement of major mediastinal vessels and extrinsic compression over the oesophagus posteriorly and LA anteriorly. No other abnormality of the tracheo-bronchial tree was noted.

**Figure 3:**
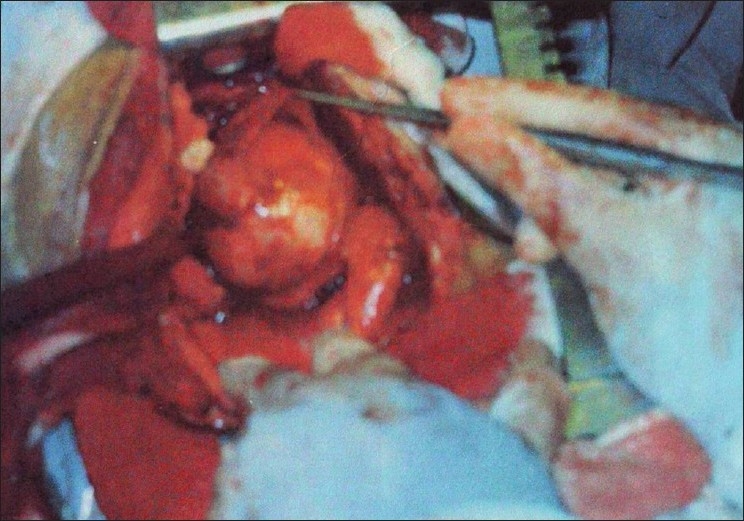
Intra-operative view of large smooth walled cystic lesion of 9.5 × 6.5 × 6 cm found in the infracarinal region of the middle mediastinum

Patient was taken for a planned surgery. A sixth space postero-lateral thoracotomy was done. A large smooth walled cystic lesion of 9.5 × 6.5 × 6 cm was found in the infra-carinal region of the middle mediastinum [Figure 3]. There was no obvious lymphadenopathy.

**Figure 4:**
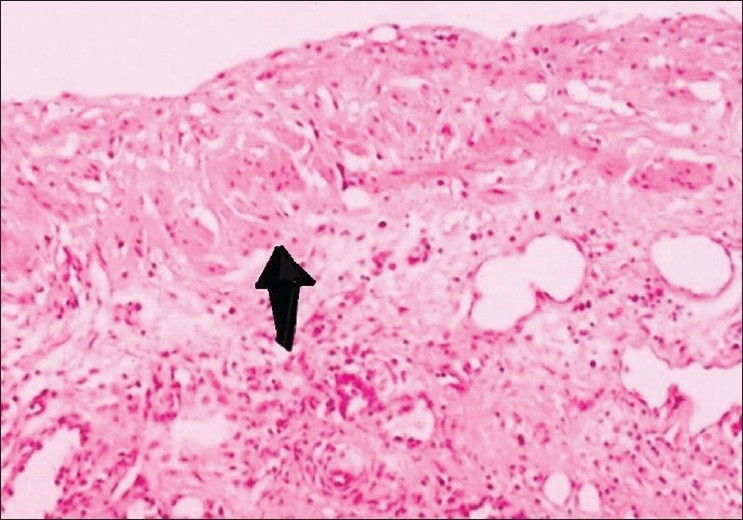
Photomicrograph (H and E stain, original magnification ×25) of the cyst wall showing absence of epithelial lining with presence of discrete smooth muscle bundles in the cyst wall (arrows) suggesting a bronchogenic origin for the cyst

The tumour was found to be displaying the two principle bronchi behind the left atrium pushing the latter anteriorly. The tumour extended almost equally on either side of the midline and was found compressing the mid-oesophagus posteriorly. Right vagus was firmly adhering to the lesion as it ran over the tumour wall. An attempt was made to dissect the cyst from the surrounding structures but was abandoned due to dense adhesions and immense vascularity. A milky white fluid on needle aspiration established the cystic nature of the tumour. A major portion of the cyst wall was excised and representative tissue was sent for histopathological examination. The remaining portion of the cyst was ablated with electrocautery. Thorax was closed and two drains were left in the thoracic cavity. The patient was extubated on the OT Table and had an uneventful recovery and was discharged on the 10^th^ post-operative day after suture removal. Histological evaluation confirmed the diagnosis of bronchogenic cyst [Figure 4].

## DISCUSSION

Bronchogenic tumours are rare tumours in the mediastinum. These are the most common primary cysts. Of the mediastinal bronchogenic cysts, 79% are located in the middle mediastinum, 17% in the posterior mediastinum, and three per cent in the anterior mediastinum.[1]

They originate as sequestration from the ventral foregut, the antecedent of the tracheo-bronchial tree. The cyst may lie within the lung parenchyma or the mediastinum. The cyst wall is composed of cartilage, mucous glands, smooth muscles and fibrous tissue with the pathognomic inner layer of ciliated respiratory epithelium. Almost 75% cases are asymptomatic. Symptoms vary with age at presentation and up to a large extent depend upon the size and location of cyst. Common symptoms are chest pain (22%), dyspnea (12%), cough (7%), stridor (7%), respiratory compromises due to tracheal/ bronchial compression and recurrent pneumonia (10%).[1,2] Unusual presentation includes dysphagia (one per cent), Pneumothorax (one per cent), Superior vena cava syndrome (one per cent).[1]

The presented case is rare in terms of mode of presentation which was dull backache and dysphagia. Backache in these tumours is caused by stretching of the nerves supplying the parietal pleura.[3] Dysphagia is a confusing symptom which initially directs the attention towards seeking a primary GI pathology. GB calculi, however, in this case turns out to be an incidental finding though a theoretical possibility of presence of diverticuli in the gall bladder is an inciting factor and the association of the two factors on an embryological basis cannot be ruled out. ECG findings are striking with non-specific ST depression and T-wave inversion in most leads. Chest X-ray findings suggested the possibility of a mass lesion in mediastinum, though, non-specific. An X-ray of the vertebral column was normal and negated the possibility of mass being neuroenteric cyst. The size of the cyst was unusual and helped in early diagnosis of the condition. Though Barium swallow pointed to an extrinsic compression of mid-oesophagus, contrast-enhanced chest CT scan clinched the diagnosis and helped in taking the decision of surgical treatment. Literature suggests endoluminal sonography and MRI as other definitive modalities and various studies have found MRI to be highly sensitive and specific in the diagnosis of this condition.[2,4] An unusual and clinically confusing presentation like this demands a thorough and detailed evaluation of such patients keeping in mind the various possibilities.

Appropriate treatment of patients with bronchogenic cysts depends on the patient's age and symptoms at presentation. Symptomatic cysts should be resected (either at thoracotomy or by means of video-assisted thoracoscopy) regardless of patient age unless surgical risks are unacceptably high. It is generally recommended that asymptomatic cysts in young patients be removed because of the low surgical risk and the potential risk of late complications (albeit rare) such as infection, hemorrhage, or neoplasia within the cyst. Conservative (watch-and-wait) treatment has been advocated in asymptomatic adults or other high-risk patients. Percutaneous catheter drainage or sterile alcohol ablation has been performed in selected cases. In such patients, radiologic diagnosis is of great importance as tissue confirmation will not be obtained.

Intra-operative findings demand special attention to avoid injury to the vagus, the tracheo-bronchial tree, pericardium and the oesophagus. Minimal possible damage to oesophageal layers has been recommended and is logical. Ablation of the cyst rules out the possibility of recurrence which, though unusual, have been reported. Thoracoscopic/videoscopic resection[5] of bronchogenic cyst is a recent innovation and should be sought whenever infrastructure and expertise is available though adherence to the left atrium rules it out in our case.

## CONCLUSION

This case study highlights dysphagia and back pain as unusual, presenting symptoms of mediastinal bronchogenic cysts, and emphasises the importance of contrast-enhanced chest CT scan in dealing with all thoracic tumours. Surgical option should be exercised as a definitive procedure in all cases to avoid development of complications.
